# Human genetic variation determines 24-hour rhythmic gene expression and disease risk

**DOI:** 10.21203/rs.3.rs-4790200/v1

**Published:** 2024-08-05

**Authors:** Dongyin Guan, Ying Chen, Panpan Liu, Aniko Sabo

**Affiliations:** Baylor College of Medicine

## Abstract

24-hour biological rhythms are essential to maintain physiological homeostasis. Disruption of these rhythms increases the risks of multiple diseases. The biological rhythms are known to have a genetic basis formed by core clock genes, but how individual genetic variation shapes the oscillating transcriptome and contributes to human chronophysiology and disease risk is largely unknown. Here, we mapped interactions between temporal gene expression and genotype to identify quantitative trait loci (QTLs) contributing to rhythmic gene expression. These newly identified QTLs were termed as rhythmic QTLs (rhyQTLs), which determine previously unappreciated rhythmic genes in human subpopulations with specific genotypes. Functionally, rhyQTLs and their associated rhythmic genes contribute extensively to essential chronophysiological processes, including bile acid and lipid metabolism. The identification of rhyQTLs sheds light on the genetic mechanisms of gene rhythmicity, offers mechanistic insights into variations in human disease risk, and enables precision chronotherapeutic approaches for patients.

## One Sentence Summary

Rhythmic quantitative trait loci explain individual variations in biological rhythms of gene expression and contribute to disease risk in humans.

## Introduction

Biological rhythms refer to recurring physiological processes with a periodicity of approximately 24 hours. These rhythms allow mammals to anticipate daily environmental changes, including light-dark cycles, and are essential to maintain physiological homeostasis^1–3^. The disruption of biological rhythms is increasingly recognized as a risk factor for multiple diseases, including type 2 diabetes, cardiovascular disease, digestive disease, and cancer^4–6^. It is generally accepted that variations of the 24-hour rhythms regarding gene expression and physiological processes exist in different individuals. Nevertheless, the major questions remain as to whether and how these variations in biological rhythms contribute to the disease risk and what the underlying mechanisms responsible for these variations.

Moreover, our current understanding of mechanisms regulating rhythmic gene expression highlights the regulatory roles of transcription factors (TFs) on *cis*-regulatory elements (CREs), including core clock components such as BMAL1 and REV-ERBs, as well as noncanonical clock TFs^7–9^. Genetic or environmental perturbation of these regulatory TFs in animal models can cause or exacerbate multiple diseases^10,11^. Variants within CREs have been linked to variations in human gene regulation, sleep disorders, individual chronotypes, and other complex traits and diseases^12–14^. However, the relationships between genetic variation, human biological rhythms in specific tissues, and the mechanisms underlying complex traits and diseases in humans are largely unknown. Since direct genetic manipulation is impractical in humans, we studied natural genetic variation in the Genotype Tissue Expression (GTEx) Project to identify associations with perturbations in gene rhythmicity and to further determine the relationship between gene rhythmicity and human phenotype in various tissues.

**Figures 1:**
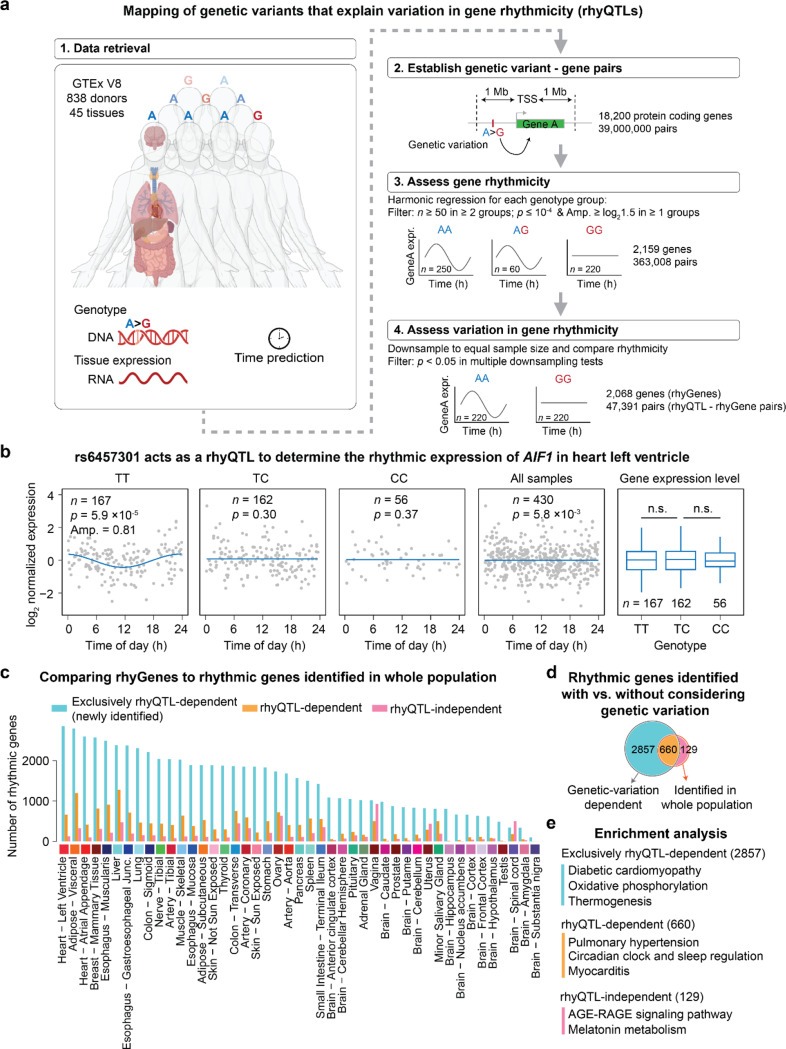
Genome-wide mapping of rhyQTLs across human tissues. **a**, Study design for rhyQTL mapping and rhyGene identification. The numbers of genes and gene-genetic variation pairs displayed in the plot represent the median values across 45 solid tissues used in this study. **b**, SNP rs6457301 as a representative rhyQTL determines the rhythmic expression of *AIF1* in the subpopulation with TT genotype but not in other genotypes. The parameters of *p* value and amplitude (log_2_ fold change of peak-to-trough) by harmonic regression fit are shown. The expression levels of *AIF1* among the three genotypes showed no statistical significance. **c**, Overlap between rhythmic genes identified with and without considering rhyQTLs across 45 tissues. **d**, Venn diagram for rhyQTL-dependent rhythmic genes and rhythmic genes identified from whole population without considering genetic variation in the left ventricle heart. **e**, Pathway enrichment analysis on exclusively rhyQTL-dependent, rhyQTL-dependent, and rhyQTL-independent rhythmic genes in the left ventricle heart.

We first established a median of 39 million genetic variant-gene pairs across 45 tissues from 838 individuals using GTEx data (**Supplementary Table 1**)^15^ and then assessed the association between genetic variants and rhythmic expression of their paired genes (Fig. 1a). Using harmonic regression to evaluate the gene rhythmicity in a subpopulation with specific genotype for each genetic variant-gene pair, we identified a median of 2,159 genes across 45 tissues that are rhythmically expressed in at least one genotype subpopulation. A median of 2,068, accounting for 95.8% of total rhythmic genes across tissues, exhibits differential rhythmic expression among genotypes (Fig. 1a). For example, the expression of allograft inflammatory factor 1 (*AIF1*) in heart tissue, which correlates with the development of cardiac allograft^16,17^, is only rhythmic in the subpopulation with TT genotype at single nucleotide polymorphism (SNP) rs6457301 (Fig. 1b). Note that the overall expression levels of *AIF1* have no difference across these three genotypes. This result indicates the relationship between SNP rs6457301 and *AIF1* rhythmicity can not be explained by expression quantitative trait loci (**eQTLs**), which reflects gene expression levels across different genotypes. Here, we name this genetic variation that explains variations in 24-hour rhythmic gene expression as rhythmic quantitative trait loci (**rhyQTLs**) and name the rhyQTL-associated rhythmic gene as **rhyGene**.

Since the rhythmic expression pattern in a subpopulation with a specific genotype could be masked by the non-rhythmic expression of the same gene in subpopulations with other genotypes, the rhythmic expression of a single gene may only be observed in a subpopulation with a specific genotype but not in the whole population. Indeed, our rhyQTL analysis uncovered a median of 3.6 times as many rhythmic genes across 45 tissues than previously recognized in whole human populations without consideration of genetic variation^18^ (Fig. 1c). For example, in heart tissue, 2,857 rhythmic genes are newly identified, displaying rhythmic expression exclusively in specific genotypes. Even in 789 genes whose rhythmicity is detected from whole populations, 84% of them are affected by rhyQTLs, and only 16% of rhythmic genes from the whole population are independent of *cis*-regulation by nearby genetic variants (Fig. 1d). To distinguish the functional importance between genetic variation-dependent and independent rhythmic genes, we performed pathway enrichment analysis. Diabetic cardiomyopathy and oxidative phosphorylation pathways are enriched in rhythmic genes that are specifically associated with rhyQTLs. The receptor for advanced glycation endproducts (RAGE) signaling pathway, which is related to diabetes-mediated vascular calcification^19^, and melatonin metabolism are enriched in rhyQTL-independent rhythmic genes (Fig. 1e). Thus, these data indicate the crucial *cis*-regulatory role of genetic variations in modulating rhythmic gene expression.

**Figures 2:**
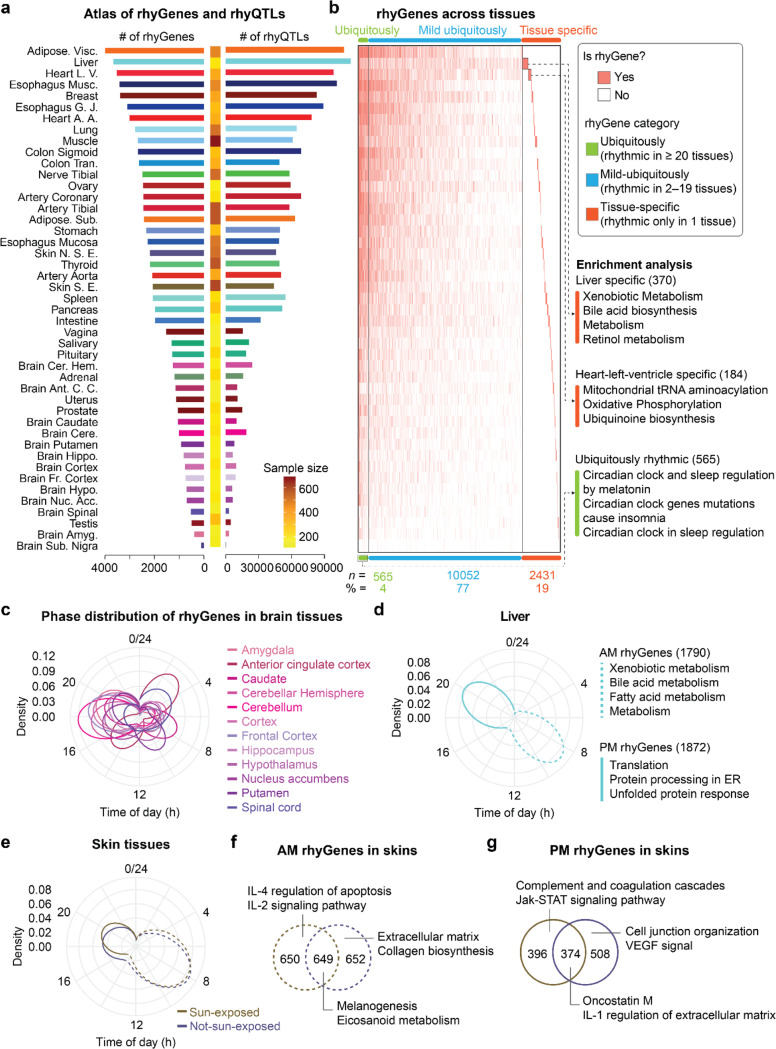
Characterization of rhyGenes. **a**, Numbers of rhyQTLs and rhyGenes in 45 tissues. The heatmap in the middle indicates the sample size with both genotype and expression data. **b**, Tissue-specific feature of rhyGenes. rhyGenes are clustered into three categories (ubiquitously rhythmic, mild ubiquitously rhythmic, and tissue-specific rhythmic) and followed by pathway enrichment analysis. **c-e**, Polar plot illustrating the peak phase distribution of rhyGenes in brain (**c**), liver (**d**), and skin (**e**) tissues. The rhyGenes with phases within 0:00 ~ 12:00 are defined as morning (AM) rhyGenes (indicated by dashed lines), while those with phases within 12:00 ~ 24:00 are defined as afternoon (PM) rhyGenes (indicated by solid lines). The skin exposed to the sun is sampled from the lower leg, while the skin not exposed to the sun is sampled from the suprapubic area. **f-g**, The overlap of AM rhyGenes (**f**) and PM rhyGenes (**g**) between sun-exposed and non-sun-exposed skin and followed by pathway enrichment analysis.

Across different tissues, we observed varying numbers of rhyQTLs and rhyGenes, including 3,996 rhyGenes and 107,883 rhyQTLs in adipose visceral tissue, and 102 rhyGenes along with 243 rhyQTLs in substantia nigra (Fig. 2a, **Extended Data Fig. 1** and **Supplementary Table 2**). To evaluate the performance of our rhyQTL detection method with a sample size of 838 individuals, we conducted two analyses. First, we assessed the power of rhyQTL discovery by randomly selecting subsets of samples of various sizes (**Extended Data Fig. 2** and detailed information in Methods). Second, we validated the rhyQTLs identified in the GTEx dataset using diurnal RNA-seq data (**Extended Data Fig. 3** and detailed information in Methods). The results indicate that the datasets from GTEx are effective in detecting genuine SNPs associated with rhythmic expression variations in humans.

To characterize the genes affected by rhyQTLs, we first quantified the number of tissues where the gene is detected as rhyGene and identified 565 rhyGenes that are common in more than 20 tissues. These common rhyGenes are related to general circadian functions, including melatonin, insomnia, and sleep regulation (Fig. 2b). Interestingly, tissue-specific rhyGenes were intricately linked to the distinctive functions of the tissue (Fig. 2b). For example, bile acid synthesis pathway and xenobiotic metabolism are enriched in liver-specific rhyGenes, while mitochondrial tRNA aminoacylation pathway is enriched in heart-specific rhyGenes.

Next, we explored whether the peak expression of the identified rhyQTLs is enriched at specific times of the day using phase analysis. We found that the phase of the rhyGenes is enriched around 5 AM or 5 PM in brain tissues (Fig. 2c and **Supplementary Table 3**). Coinciding with the concept that brain clocks act as a pacemaker to synchronize biological rhythms in peripheral tissues^20^, peripheral tissues show a 2 to 6-hour delay in their phases (Fig. 2d, 2e and **Extended Data Fig. 4**). To determine which physiological processes are regulated by these rhyGenes, we performed pathway enrichment analysis and found that xenobiotic, bile acid, and fatty acid metabolism are enriched in hepatic rhyGenes with peak expression in the morning, while protein translation and unfolded protein response are enriched in hepatic rhyGenes with peak expression in the afternoon (Fig. 2d). Intriguingly, the phases in humans regarding metabolism and stress pathways exhibit a 12-hour shift compared to these in nocturnal mouse models ^21^. These morning and evening waves of rhyGenes are not directly regulated by sunlight exposure because the rhyGenes in sun-exposed and not-sun-exposed skin tissues show similar phase distribution (Fig. 2e). Although the expression waves are similar, the rhyGenes are different in that cell adhesion and metabolism pathways are enriched in not-sun-exposed skin, while inflammation and immuno-response pathways are enriched in sun-exposed skin (Fig. 2f and 2g). Together, these results indicate that rhyQTLs are associated with a wide range of rhythmic gene expression in various tissues to regulate time-dependent physiological processes.

**Figures 3:**
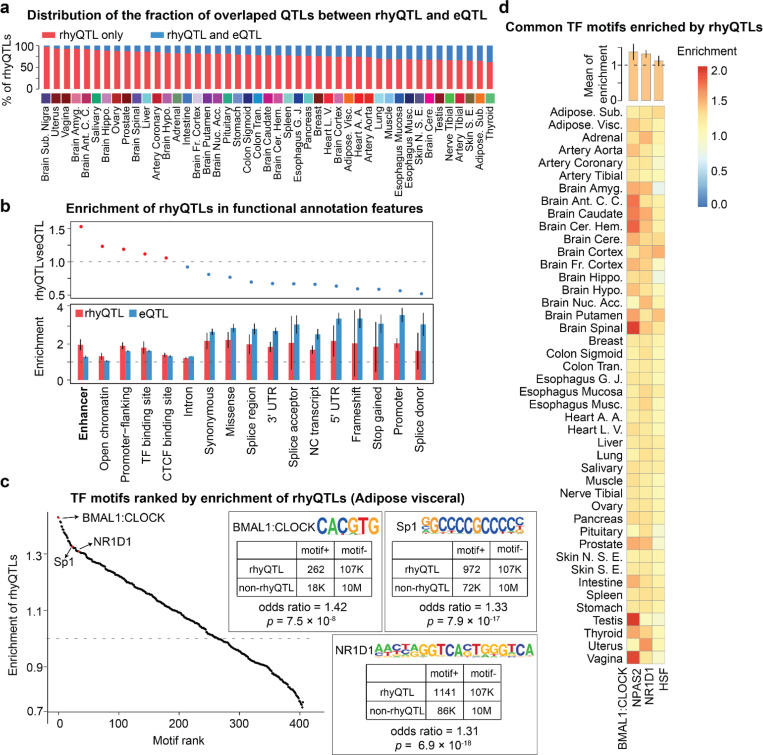
rhyQTLs represent a novel type of molecular QTL. **a**, The overlap of genomic loci between rhyQTLs and eQTLs. **b**, Enrichment of QTLs in genomic annotation categories defined by Ensembl VEP. Each bar represents the median of enrichment in each category across 45 tissues, with error bars representing the standard deviation across tissues. The enrichment is calculated as an odds ratio score using the numbers of observed QTLs and expected QTLs located in each annotation category compared to those in baseline regions. Baseline regions are unions of all functional and putative functional regions within the human genome. **c**, TF motif enrichment in genomic loci harboring rhyQTLs from visceral adipose tissue. The enrichment of TF was calculated using 2 × 2 contingency tables. The tables of representative motifs were shown along with their corresponding motif logos.*p* value was calculated using Fisher’s exact test. In the scatter plot, each dot represents a TF motif. **d**, The heatmap of enrichment of the most enriched motifs across 44 tissues with the number of rhyQTLs greater than 2000. The bars on the top indicate the mean enrichment across tissues, with error bars representing the standard deviation across different tissues.

To further dissect the potential distinct or combinational effects on gene rhythmicity and abundance contributed by genetic variation, we calculated the proportion of rhyQTLs that are also eQTLs and found that only 3% to 38% of rhyQTLs in various tissues are also eQTLs (Fig. 3a). These results indicate rhyQTLs are largely different from eQTLs and majorly regulate gene rhythmicity but not overall expression level. To compare the molecular mechanisms of identified rhyQTLs relative to eQTLs, we performed enrichment analyses using the annotation genomic functional elements defined by VEP^22^. We found that although both eQTLs and rhyQTLs are enriched in various gene regulatory regions (odds ratio > 1), rhyQTLs are more enriched in enhancer regions compared to eQTLs (Fig. 3b and **Supplementary Table 4**). This is consistent with the findings that clock regulators prefer binding to enhancer regions in mouse models^21,23^, suggesting rhyQTLs contribute to variations in enhancer activities for regulating target rhyGenes.

Emerging studies indicate that QTLs convert genetic information to its function by alternating TF DNA-binding consensus sequence and affecting TF chromatin binding affinity^14,24^. To identify which TFs may be regulated by this mechanism, we scanned putative TFs that bind to rhyQTLs by cross-referencing rhyQTLs with the known human TF motifs. We found that binding motifs of core clock genes, such as BMAL1, CLOCK, and NR1D1 were highly enriched in rhyQTLs from adipose tissue, aligning with their roles in rhythmic gene expression. Additionally, the binding motifs of TFs that are functional in adipose tissue, such as SP1, which relates to adipose lineage differentiation^25^, was found to be enriched in adipose rhyQTLs (Fig. 3c). Notably, the enrichment of binding motifs for core clock genes was observed across most tissues (Fig. 3d), indicating an interaction between core clock genes and genetic variations for regulating the variation of biological rhythms.

**Figures 4:**
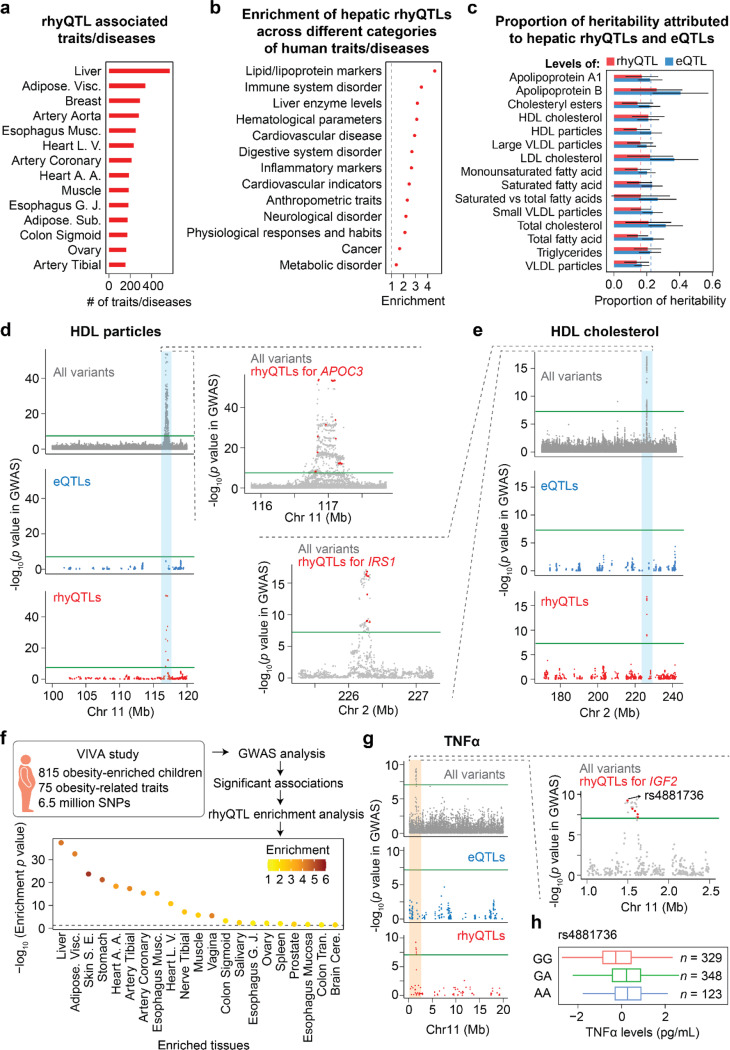
rhyQTLs contribute to human traits/diseases. **a**, The counts of rhyQTL-associated traits/diseases. Tissues with rhyQTL-associated traits/diseases ≥ 150 are displayed. **b**, Enrichment of hepatic rhyQTLs in the GWAS SNPs associated with different categories of human diseases/traits. The enrichment is calculated as the odds ratio score based on a 2 × 2 contingency table that includes the count of SNPs classified by whether they are rhyQTLs and whether they are GWAS-tag SNPs. Fisher’s exact test was used to estimate the *p* value. **c**, Partitioned heritability plot for the proportion of phenotypic variance that can be explained for 15 traits by rhyQTLs and eQTLs. The proportion of heritability is quantified as the ratio of heritability attributed to rhyQTLs or eQTLs to the overall SNP-based heritability using stratified linkage disequilibrium score regression (LDSC). The red and blue dashed lines represent the median values of rhyQTL and eQTL across traits, respectively. Error bars indicate the 95% confidence interval (CI) of the estimate. **d-e**, Representative examples demonstrate that traits are associated with rhyQTLs rather than eQTLs. The top track shows the -log_10_(*p*) values of SNPs from the GWAS of HDL particles (**d**) and HDL cholesterol (**e**). The middle and bottom tracks display the -log_10_(*p*) values in the GWAS of eQTL SNPs (middle track) and rhyQTL SNPs (bottom track) of all protein-coding genes within the region, respectively. **f**, Enrichment of rhyQTLs among significant GWAS SNPs identified in an obesity-enriched cohort from VIVA study. Tissues with an odds ratio > 1 and *p* value < 0.05 in Fisher’s exact test are shown and ranked based on their enrichment *p* values. The color of the dots indicates the odds ratios in the respective tissues. **g**, TNFα levels in the population enriched for obesity are associated with rhyQTLs rather than eQTLs. **h**, The TNFα levels among various genotype subpopulations for the SNP rs4881736.

Genetic variants, especially those in CREs, have been linked to human complex traits and diseases through GWAS. To decipher whether rhyQTLs can explain the variations in human traits and diseases, we retrieved around 500,000 published SNP-trait/disease associations from GWAS Catalog^26^ and assessed the enrichment of rhyQTLs within these GWAS-tag SNPs across different tissues (**Extended Data Fig. 5a**). We found that rhyQTLs show more statistical significance in GWAS than control SNPs (**Extended Data Fig. 5b**) and hepatic rhyQTLs explain the most traits/diseases (Fig. 4a and **Supplementary Table 5**). To further dissect the specific traits/diseases that were contributed by hepatic rhyQTLs, we classified the traits/diseases from GWAS Catalog into specific physiological function or disease-related categories and then calculated the enrichment of these rhyQTLs in each category (**Extended Data Fig. 5a** and **Supplementary Table 6**). We observed that hepatic rhyQTLs are enriched across all categories of traits/diseases (odds ratio > 1), with the top enriched traits related to lipid/lipoprotein markers, which directly reflect liver-related functions (Fig. 4b).

Heritability refers to the proportion of variation in a population trait that can be attributed to inherited genetic factors^27^. To further explore whether the heritability of lipid/lipoprotein-related traits is attributed to rhyQTLs, we conducted partitioned heritability for 15 lipid or lipoprotein-related traits using stratified linkage disequilibrium score regression (LDSC)^28–30^. Among these traits, rhyQTLs and eQTLs explained a median of 16.3% and 22.5% SNP heritability, respectively (Fig. 4c). Notably, the proportion of heritability attributed to rhyQTLs in certain traits was comparable to that of eQTLs, such as in traits related to high-density lipoprotein (HDL) cholesterol and triglyceride levels (Fig. 4c). Thus, these results demonstrate that rhyQTLs extensively contribute to human complex traits and diseases, with the most traits being associated with the liver functions.

To estimate the causality of specific rhyQTLs to specific human phenotypes, we identified rhyQTLs that share the same causal variants with significant GWAS signals in 55 lipid/lipoprotein and liver-enzyme related traits. We found that 296 GWAS signals for these traits shared their causal genetic variations with rhyQTLs. Among these, 106 GWAS signals exclusively exhibited rhyQTL signals, with no eQTL signals detected in the corresponding genomic regions (**Supplementary Table 7**). For example, apolipoprotein C3 (*APOC3*) and insulin receptor substrate-1 (*IRS1*) have been reported to regulate HDL particle levels and HDL-cholesterol levels ^31–33^. Here, we found a co-occupancy between rhyQTLs of *APOC3* and *IRS1* and genetic variants contributing to these two metabolic traits (Fig. 4d and 4e), while no eQTL was found in the same regions. These results suggest that genetic variants mediate the rhythmicity of *APOC3* and *IRS1*, rather than their overall expression levels, and contribute to the levels of HDL particle and HDL-cholesterol, respectively.

Recent studies indicated that disruption of 24-hour rhythms can exacerbate metabolic disorders in obese animal models^8,34^. To further determine whether our identified rhyQTLs could cause or even magnify the variation in metabolic phenotypes among obese individuals, we performed GWAS analysis on 75 obesity-related traits in a cohort containing 815 children enriched for obesity(**Supplementary Table 8**). Our analysis identified 186 SNP-trait associations (**Supplementary Table 9**) and found that rhyQTLs in multiple metabolic tissues, including liver, stomach, and adipose tissues, contribute to the above obesity-related traits (Fig. 4f). Notably, a GWAS signal on chromosome 11 for TNFα levels was detected specifically in this population but was not present in the general population (Fig. 4g and 4h). In this region, rhyQTLs of *IGF2*, a gene that relates to TNFα level^35,36^, are detected, while no eQTLs are observed (Fig. 4g), suggesting that the rhythmic expression variances of *IGF2* may play a role in TNFα levels, in the obese population.

By analyzing genome-wide genotypes and nearby gene rhythmicity, we mapped a new type of QTL, termed rhyQTL, that determines the variations in rhythmic gene expression in various human tissues. Besides *cis*-effects, we also detected the *trans*-effects of rhyQTLs by using core clock gene *NR1D1*-associated rhyQTLs and indicated the *trans*-regulatory role of rhyQTLs on distal gene rhythmicity (**Fig.S6** and detailed information in Methods). rhyQTLs provide novel insights into the variation in chronophysiology among individuals, such as bile acid biosynthesis and xenobiotic metabolism. The genomic loci of rhyQTLs are largely different from eQTLs in terms of genomic coordinates and regulatory mechanisms. Compared with eQTL, rhyQTLs are more enriched in enhancer regions, which fits the general concept that the gene rhythmic expression is interdependent on environmental cues that mediate enhancer activity and intrinsic clock machine^7,21,23^. Regarding the functional importance of rhyQTLs and variations in gene rhythmicity, we have observed that rhyQTLs (independent of eQTLs) extensively contribute to variations in human disease risk, including digestive system disorders and cardiovascular diseases. Taken together, rhyQTLs-mediated variations in rhythmic gene expression provide a different angle to explain GWAS signals, additional considerations on genetic variation for understanding chronophysiology in humans, and rationales for optimizing chronotherapy based on patients’ genetic backgrounds.

## Methods

### Data retrieval and data preprocessing for rhyQTL mapping

(**Step 1 in** Fig. 1a). The genotype data of 838 individuals in GTEx project were downloaded from dbGaP with accession number phs000424.v8.p2. To identify rhyQTLs, we used PLINK ^37^, a tool set for whole-genome association studies, to filter genetic variants with standard quality control criteria ^38^. We retained genetic variants with a minor allele frequency (MAF) ≥ 0.01, Hardy-Weinberg Equilibrium (HWE) *p* ≥ 10^− 6^ and located on autosomes. To gain high-confident rhythmic gene expression with sufficient samples, tissues with more than 100 samples were retained, as indicated in **Supplementary Table 1** Normalized gene expression data for retained 45 tissues of 838 individuals were downloaded from Zenodo: https://zenodo.org/records/7215362 ^18^. The data were preprocessed by using a linear regression model on log-transformed normalized count data, which regressed out several covariates explaining significant portions of the variance. These covariates, including ischemic time, sex, age, and type of death, were treated as categorical variables. The genomic annotation data (gencode.v26.GRCh38.genes.gtf) in which isoforms were collapsed to a single transcript per gene based on the GENCODE 26 annotation were downloaded from GTEx portal (https://gtexportal.org/home/).

### Establish genetic variant-gene pairs and assess the rhythmicity for rhyQTL mapping

(**Step 2 and 3 in** Fig. 1a). To determine the *cis*-regulatory effects of genetic variation on gene rhythmic expression, *cis*-genetic variants within the transcription start site (TSS) +/− 1 Mb flanking region of each of the protein-coding genes were used to establish the genetic variant-gene pairs. This 1 Mb distance is commonly employed to identify diverse types of molecular *cis*-QTLs ^39–41^. We established a median of 39 million genetic variant-gene pairs across 45 tissues with approximately 2.9 thousand *cis*-genetic variants per gene. Moreover, for robust statistical analysis, genetic variant-gene pairs with sample sizes greater than 50 in two or three subpopulations with different genotypes were retained to evaluate the gene rhythmicity in each genotype using harmonic regression ^18^. If genes met the criteria of *p* value ≤ 10^− 4^ and a fold change of peak-to-trough ≥ 1.5 in at least one genotype, the gene-genetic pair was retained as a union of possible rhyQTLs and rhyGenes.

### Assess variations in gene rhythmicity for rhyQTL mapping

(**Step 4 in** Fig. 1a). To map the genetic variants that associate with variations in gene rhythmicity, a Bayesian information criterion (BIC) based model-selection algorithm, *dryR* ^42^, was used to distinct the differential rhythmicity of genes in each genetic variant-gene pair across different genotypes. To mitigate biases associated with sample size, a downsampling approach was conducted to ensure equal sample sizes across compared genotypes. To minimize the loss of sample size during downsampling, the top two genotype groups with the most samples were included to assess the differential gene rhythmicity. Specifically, for each genetic variant-gene pair, the sample size for each of the three genotypes (for example, SNP rs6457301 has three genotypes: TT, TC, and CC) was determined, and the top two genotype groups with the largest sample sizes were selected. The genotype group with the more samples was subsampled to match the sample size of the smaller group, ensuring the equal sample sizes between the two compared genotypes. Subsequently, the differential rhythmicity was evaluated using *dryR* ^42^, which provides a fitted model to categorize the differentiation in rhythmicity between the two compared genotypes. All parameters for 24-hour rhythms, including rhythmicity, phase shift, and amplitude change, were considered for evaluating the association between genetic variants and rhythmic gene expression.

To mitigate bias related to single-time sampling, we performed twenty times of downsampling for *dryR* analysis and then used *G*-test to determine if the frequency of the fitted model provided by *dryR* across the twenty times significantly deviated from the expected frequency. Genetic variant-gene-pairs with *p* value < 0.05 in *G*-test were identified as rhyQTL-rhyGene pairs. Thus, a genetic variation was defined as a rhyQTL based on the following criteria: (1) its paired gene exhibited a rhythmic expression pattern in at least one genotype subpopulation with a peak-to-trough ratio ≥ 1.5 and *p* value ≤ 10^− 4^; (2) rhythmic expression patterns of its paired gene vary across genotypes assessed by *dryR*; (3) the observed differential rhythmicity was significantly different from the expectation in *G*-test (*p* < 0.05).

### Evaluate performance of rhyQTLs mapping strategy using downsampling analysis.

To evaluate the performance of our rhyQTL detection method with a sample size of 838 individuals, we compared the power of rhyQTL and rhyGene discovery across various sample sizes by randomly selecting samples in adipose, which contains the highest number of rhyQTLs. Samples were randomly selected from 469 adipose visceral tissue samples to create 8 data subsets. The sample sizes in each subset range from 100 to 450 at intervals of 50. In each data subset, genetic variation-gene pairs are established, and gene rhythmicity is assessed using the same methods as in “Establish genetic variant-gene pairs and assess the rhythmicity for rhyQTL mapping” section. To evaluate and mitigate biases associated with sample size, variations in gene rhythmicity across different genotypes were assessed both with and without employing a downsampling strategy. We found that when the sample size exceeded 250, there were mild changes in the numbers of identified rhyQTLs and rhyGenes, which suggests that the current sample size can robustly detect rhyQTLs and rhyGenes.

### Evaluate rhyQTLs identified in the GTEx dataset using diurnal RNA-seq data.

To validate and evaluate our rhyQTL detection method with a sample size of 838 individuals, we compared the rhyQTLs identified from GTEx samples with publicly available diurnal RNA-seq data. This dataset was derived from vastus lateralis muscle biopsies collected from 10 healthy donors every 4 hours across a day ^43^. The temporal expression levels at pre-mRNA (intronic signal) and mRNA (exonic signal) were quantified and gene rhythmicity was estimated using harmonic regression across 10 donors. Genes exhibiting rhythmicity at both pre-mRNA and mRNA levels in at least two donors (*p* < 0.05 and a peak-to-trough fold change > 1.5) were included in the set of potential rhythmic genes in this cohort. To evaluate rhyQTLs identified in GTEx dataset, genetic variations that occurred in these 10 donors in the transcription region were called based on the reads in RNA-seq data using the Genome Analysis Toolkit (GATK) ^44^, following the pipeline designed for germline SNP calling from RNA-seq data (https://github.com/gatk-workflows/gatk4-rnaseq-germline-snps-indels). Due to the small sample size of this cohort, genetic variations with a sample size greater than two in at least two genotype groups were used to intersect with rhyQTLs identified from the GTEx muscle samples. As a result, we identified a total of 1,410 rhyQTL-rhyGene pairs, comprising 1,397 rhyQTL SNPs and 292 associated rhyGenes, for the following comparison between the two cohorts. We observed 760 rhyQTL-rhyGene pairs, comprising 754 rhyQTLs and 225 associated rhyGenes, exhibit differential rhythms regarding rhythmicity (*p* value < 0.05), amplitude (fold change > 1.5) or phase (peak phase > 3 hours) ^45^ between top two genotypes with the most samples. Therefore, 54% of the rhyQTLs and 77% of the rhyGenes could be validated in this 10 individuals cohort. Notably, among the 1,397 rhyQTLs, 38% of them were associated with rhyGenes that did not exhibit rhythmicity among all three genotypes in the validation dataset. The lack of rhythmicity of these genes could be due to the limited number of samples. Considering the nature of the validation dataset with 10 donors, the proportion of validated rhyQTLs and rhyGenes could be higher with a larger sample size. This also highlights the power of GTEx data for exploring the interactions between genetic variation and rhythmic gene expression within the currently available datasets.

### Enrichment of rhyQTL and eQTL for genomic regulatory elements.

The genomic annotation categories defined by the Variant Effect Predictor (VEP) for each SNP were downloaded from GTEx portal. QTL (rhyQTL and eQTL) enrichment in each annotation category was calculated referring to the methodology outlined by Rozowsky et al. ^46^. In brief, an odds ratio score was calculated based on a 2 × 2 contingency table, including the numbers of observed QTLs and expected QTLs located in the element compared to those in the baseline regions.

$$Enrichment=\frac{E_{\mathrm{Obs}}/E_{Exp}}{B_{\mathrm{Obs}}/B_{Exp}}$$

in which $E_{Obs}$ and $E_{Exp}$ are the number of observed QTLs and the expected QTLs in the annotation category, respectively; $B_{\mathrm{Obs}}$ and $B_{Exp}$ are the number of observed QTLs and the expected QTLs in the baseline region. The baseline regions encompass a comprehensive union of all functional and putative functional regions within the human genome. These functional regions include coding regions, untranslated regions, non-coding RNA genes, open chromatin regions, TF binding sites, as well as active and repressed histone peaks derived from various tissue and cell types, along with evolutionarily conserved regions ^47^. To generate the expected set of QTLs, we use number-matched randomly selected SNPs with the same MAF distribution as observed QTLs in each tissue. Random sampling was repeated 30 times, and the median value of the odds ratios from these 30 iterations is considered as the enrichment value for each tissue.

### Transcription factor (TF) DNA binding motif scan on genomic loci harboring rhyQTLs.

We collected 436 human TF motifs from Homer database ^48^. The occurrence of each motif in human genome was scanned using FIMO (*p* value < 10^− 4^) ^49^ and then intersected with SNP loci identified from GTEx dataset using bedtools ^50^. These preprocesses established the link between the co-occurrence of TF binding motif and genetic variants at various genomic coordinates. These genetic variants were further classified by whether they were rhyQTLs and whether they were within the motif to generate a 2 × 2 contingency table for each motif. The odds ratio was used as a measure of rhyQTL enrichment in a motif, and Fisher’s exact test was applied for statistical significance (*p* < 0.05).

### GWAS enrichment analysis.

All SNP-trait/disease associations identified in GWAS were obtained from the GWAS Catalog ^51^ containing 569,163 associations. To generate a set of high-quality SNPs associated with human traits/diseases, we performed the following quality controls by removing insignificant associations (*p* values > 5 × 10^− 8^), associations obtained from non-European studies, and SNPs in the human leukocyte antigen locus (for hg38: chr6:29,723,339 − 33,087,199). After filtering, we got a clean list of 242,822 SNP-trait/disease associations in GWAS.

To explore which traits or diseases are contributed by hepatic rhyQTLs, we evaluated the enrichment of rhyQTLs among these GWAS-tag SNPs. Traits/diseases were divided into different categories according to their parent terms as per the annotation in GWAS Catalog (**Supplementary Table 6**). The set of GWAS-tag SNPs in each category was extended by including the SNPs in high linkage disequilibrium (LD scores = 1) with the tag SNPs to ensure more comprehensive coverage of potential causal variants and increase the statistical power in the enrichment analysis. The odds ratio score was calculated to determine the enrichment of hepatic rhyQTLs in each trait/disease category. The 2 × 2 table included the number of SNPs classified by whether they were rhyQTLs and whether they were GWAS-tag SNPs.

$$OR=\frac{a\times d}{b\times c}$$

where a is the number of SNPs that were both rhyQTLs and GWAS-tag SNPs, b is the number of SNPs that were rhyQTLs but not GWAS-tag SNPs, c is the number of SNPs that were GWAS-tag SNPs but not rhyQTLs, and d is the number of SNPs that were neither rhyQTLs nor GWAS-tag SNPs. Fisher’s exact test was applied for statistical significance (*p* value < 0.05).

### Stratified linkage disequilibrium score (LDSC) regression.

The stratified LDSC regression was used to quantify the heritability attributable to rhyQTLs and eQTLs for the 15 lipid or lipoprotein-related traits^30,52–54^. This approach regresses chi-square statistics from the GWAS summary statistics, which were downloaded from GWAS Catalog, with LD scores to estimate partitioned heritability in a disease-specific manner. To do this, binary annotation for rhyQTLs and eQTLs was created, respectively. This annotation assigns a value of 1 to rhyQTL or eQTL SNPs and a zero value to the remaining SNPs in the baseline regions ^47^. The LD scores of the rhyQTL and eQTL were computed using SNP genotype data of the individuals of European ancestry from the 1000 Genomes Project Phase 3 with a window size of 1 centimorgan (cM). The proportion of heritability is quantified as the ratio of heritability attributed to rhyQTLs or eQTLs to the overall SNP-based heritability.

### Finding rhyQTLs that contribute to specific human traits.

To estimate the causality of specific rhyQTLs to specific human traits, rhyQTLs that share the same putative causal variants with significant GWAS signals were identified in 55 lipid/lipoprotein-related traits (list in **Supplementary Table 7**). Firstly, lead SNPs were identified using the ld_clump function of the ieugwasr R package ^55^, which were defined as independent SNPs that had the most significant *p* value within each GWAS signal. Secondly, eQTLs and rhyQTLs for all protein-coding genes within each lead-SNP surrounding region of +/− 1 Mb were counted. If there are five or more rhyQTLs in a lead-SNP surrounding region, it was considered that the rhyQTLs contribute to the GWAS signal. Additionally, if no eQTLs are detected in this region, it was considered that the rhyQTLs contribute to the GWAS signal independently of eQTLs.

### GWAS on Viva la Familia study (VIVA) dataset.

The VIVA was designed to investigate genetic and environmental factors affecting obesity and its comorbidities in Hispanic children. Each family involved in the VIVA cohort was ascertained on a proband with obesity between the ages 4–19 years. The VIVA cohort was highly enriched for obesity: most of the parents were either classified as overweight (34%) or obese (57%), and 52% of the enrolled children were classified as obese (above the 95th BMI percentile). Among the obese children, 62% were above the 99th BMI percentile, indicating severe obesity. All participants were genotyped using marker assays included on the Illumina HumanOmni1-Quad v1.0 BeadChips. 75 obesity-related phenotypes were measured, including birth weights retrieved from Texas birth records, anthropometric and body-composition traits via dual-energy x-ray absorptiometry, dietary assessments through 24-hour recalls, total energy expenditure and substrate utilization monitored using 24-hour room calorimetry, physical activity tracked via accelerometry, and fasting biochemistries analyzed using standard techniques (listed in **Supplementary Table 8**). Subjects and study procedures are described in detail as previously ^56–58^.

GWAS on these 75 obesity-related phenotypes was conducted following the GWAS standard pipeline^59,60^. Firstly, data quality control was performed to remove SNPs and individuals with insufficient genotyping quality using PLINK v1.90b6.16 64-bit ^37^. Specifically, the SNPs with call rates < 98% or with MAF < 0.05 and those with genotypes not in accordance with the Hardy–Weinberg equilibrium (*p* > 10^− 6^) were eliminated. Individuals with a call rate of less than 98% were also excluded. Then, principal component analysis (PCA) was performed to check for population stratification using PLINK. The first 10 principal components, family structure, age, and gender information, were included as covariates in the association study. A genome-wide significance threshold of *p* was established at ≤ 7.7 × 10^− 8^ based on the number of SNPs (644,251 SNPs) included in the association analysis, and SNPs exceeding this threshold were considered as candidate variants (**Supplementary Table 9**). The enrichment of rhyQTLs in GWAS-tag SNPs identified in populations enriched for obesity was calculated based on the odds ratio score as described above in the section of GWAS enrichment analysis.

### Estimate trans-effect of rhyQTLs on rhythmic gene expression.

To determine the *trans*-effects of rhyQTLs on rhythmic gene expression, we focused on core clock genes since their relationship with target genes has been well established in animal models, including core clock gene transcription feedback loops ^61^. We first determined whether the rhythmic expression of core clock genes is associated with genetic variations in various tissues. Indeed, the rhythmic expression of core clock components, including *ARNTL*, *NR1D1* and *NR1D2*, are associated with genetic variations in multiple tissues (**Extended Data Fig. 6a**). For example, SNP rs11870683, which correlates to bipolar disorder in genome-wide association studies (GWAS) ^62^, contributes to the rhythmic expression of *NR1D1* in the subpopulations with genotype TT (**Extended Data Fig. 6b**).

Next, we evaluated the effects of *NR1D1*-associated rhyQTLs on the rhythmic expression of distal genes located either on the same chromosome or on different chromosomes (**Extended Data Fig. 6c**). We established the genetic variants and gene pairs between rhyQTLs associated with *NR1D1* rhythmic expression and all protein-coding genes. To exclude potential *cis*-effects of these rhyQTLs, we used only *trans*-genetic locus and gene pairs with a genomic distance greater than 1 Mb between the locus and the gene TSS for downstream analyses. For each pair, assessment of gene rhythmicity and variation in rhythmicity across different genotypes were conducted using methods outlined in the “Establish genetic variant-gene pairs and assess the rhythmicity for rhyQTL mapping” and “Assess variations in gene rhythmicity for rhyQTL mapping” sections. In total, we identified 489 *trans*-rhyGenes whose rhythmicity could be mediated by *NR1D1*-associated rhyQTLs in 13 brain tissues (**Extended Data Fig. 6d** and **Supplementary Table 10**). These results indicate the *trans*-regulatory role of rhyQTL on distal genes. Given that the *trans*-association is indirect and involves complex regulatory networks, we focused on *cis*-effects of rhyQTLs for high confident regulation in this study.
